# Porous Pt Nanospheres Incorporated with GOx to Enable Synergistic Oxygen‐Inductive Starvation/Electrodynamic Tumor Therapy

**DOI:** 10.1002/advs.202001223

**Published:** 2020-07-06

**Authors:** Zijie Lu, JiaYu Gao, Chao Fang, Yi Zhou, Xiang Li, Gaorong Han

**Affiliations:** ^1^ State Key Laboratory of Silicon Materials School of Materials Science and Engineering Zhejiang University Hangzhou Zhejiang 310027 P. R. China; ^2^ The Affiliated Stomatology Hospital Zhejiang University School of Medicine Key Laboratory of Oral Biomedical Research of Zhejiang Province Hangzhou Zhejiang 310027 P. R. China

**Keywords:** electrodynamic therapy, oxygen‐inductive starvation, porous Pt nanospheres, synergistic tumor therapy

## Abstract

Glucose‐oxidase (GOx)‐mediated starvation by consuming intracellular glucose has aroused extensive exploration as an advanced approach for tumor treatment. However, this reaction of catalytic oxidation by GOx is highly dependent on the on‐site oxygen content, and thus starvation therapy often suffers unexpected anticancer outcomes due to the intrinsic tumorous hypoxia. Herein, porous platinum nanospheres (pPts), incorporated with GOx molecules (PtGs), are synthesized to enable synergistic cancer therapy. In this system, GOx can effectively catalyze the oxidation of glucose to generate H_2_O_2_, while pPt triggers the decomposition of both endogenous and exogenous H_2_O_2_ to produce considerable content of O_2_ to facilitate the glucose consumption by GOx. Meanwhile, pPt induces remarkable content of intracellular reactive oxygen species (ROS) under an alternating electric field, leading to cellular oxidative stress injury and promotes apoptosis following the mechanism of electrodynamic therapy (EDT). In consequence, the PtG nanocomposite exhibits significant anticancer effect both in vitro and in vivo. This study has therefore demonstrated a fascinating therapeutic platform enabling oxygen‐inductive starvation/EDT synergistic strategy for effective tumor treatment.

## Graphical abstract

Porous platinum nanospheres (pPts) incorporated with glucose oxidase (GOx) molecules (PtGs) are synthesized for synergistic electrodynamic/starvation therapy. Despite its feasible loading for GOx, pPt enables sufficient O_2_ supply to facilitate GOx‐mediated starvation by decomposing H_2_O_2_. Meanwhile, PtG induces reactive oxygen species (ROS) under an electric field following an electrodynamic mechanism. Considerable in vitro and in vivo tumor inhibition is consequently achieved.

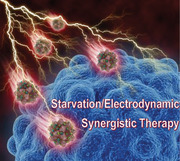

## 1 Introduction

In recent years, starvation therapy has aroused extensive attention as a burgeoning cancer treatment modality.^[^ ^1^ ^]^ It is known that cancer cells can proliferate in a rapid manner, and this requires a considerable supply of nutrients.^[^ ^2^ ^]^ The cutting‐off of nutrient supply to induce starvation may effectively inhibit tumor growth. At present, there are mainly two mechanisms involved in this strategy. One is direct cutting‐off of blood supply at the tumor regime by blocking/destroying the blood vessels or inhibiting angiogenesis.^[^ ^3^ ^]^ Another is to deplete essential nutrients in tumor.^[^ ^4^ ^]^ As a type of oxidoreductase, glucose oxidase (GOx) presents intrinsic properties in converting glucose, oxygen, and water into gluconic acid, and simultaneously hydrogen peroxide is generated.^[^ ^5^ ^]^ Given the crucial role of glucose metabolism in tumor growth, it is logic that the tumor would starve to death with direct glucose deprivation by GOx.

Nonetheless, the catalytic reaction triggered by GOx relies considerably on the presence of oxygen, and thus its enzymatic activity suffers a low efficacy from the hypoxia condition which is a characteristic hallmark of malignant solid tumors.^[^ ^6^ ^]^ Great effort has been devoted to tackling this challenge. Representative tumor reoxygenation strategies fall into three main branches: one is to directly deliver oxygen reservoirs to the tumor site using perfluorocarbon (PFC), for instance.^[^ ^7^ ^]^ The second is to utilize peroxidase or peroxidase‐mimicking nanoenzymes to break down H_2_O_2_ and produce oxygen in situ, such as MnO_2_,^[^ ^8^ ^]^ Prussian Blue,^[^ ^9^ ^]^ and catalase (CAT).^[^ ^10^ ^]^ Another is to generate oxygen by the decomposition of certain oxide or peroxide compounds (e.g., Au_2_O_3_ and CaO_2_).^[^ ^11^ ^]^ To combine the GOx‐mediated starvation with oxygen‐inductive compounds is therefore expected to promote the therapeutic efficacy by a large magnitude.^[^ ^12^ ^]^ Alternatively, the combination of GOx‐mediated starvation approach with other therapeutic modalities, including chemotherapy,^[^ ^13^ ^]^ phototherapy,^[^ ^14^ ^]^ gas therapy,^[^ ^15^ ^]^ chemodynamic therapy,^[^ ^16^ ^]^ and immunotherapy,^[^ ^17^ ^]^ has also been extensively explored aiming at the promoted antitumor outcomes.

Electrodynamic therapy (EDT) is an emerging external‐stimuli‐activated therapeutic modality.^[^ ^18^ ^]^ Its main mechanism is the on‐demand generation of reactive oxygen species (ROS) by platinum (Pt) nanoparticles (Pt NPs) under an alternating electric field. More interestingly, in the presence of chloride ions, the electric field triggers the decomposition of water molecules at the surface of Pt NPs, resulting in ROS production that could induce increased intracellular oxidative stress and initiate cell apoptosis. Owing to its strong ablation capability for tumors with relatively larger dimension, EDT has been recognized as a highly potential approach for the effective treatment of malignant solid tumor. In addition, unlike other “dynamic therapies,” the ROS production in EDT does not depend on the on‐site oxygen or H_2_O_2_ content in the tumor microenvironment. Meanwhile, it is worth noting that platinum nanoparticles, as a type of peroxidase‐like nanocatalyst, can effectively catalyze the decomposition of H_2_O_2_ to water and oxygen. Compared with MnO_2_ and CaO_2_ compounds for O_2_ generation, Pt possesses remarkably lower cytotoxicity and strong stability in a physiological environment.^[^ ^19^ ^]^ It is thus expected that the combination of Pt nanoparticles and GOx may potentially serve as an alternative therapeutic platform for EDT/starvation synergistic treatment with capability of modulating hypoxic microenvironment.

**Scheme 1:**
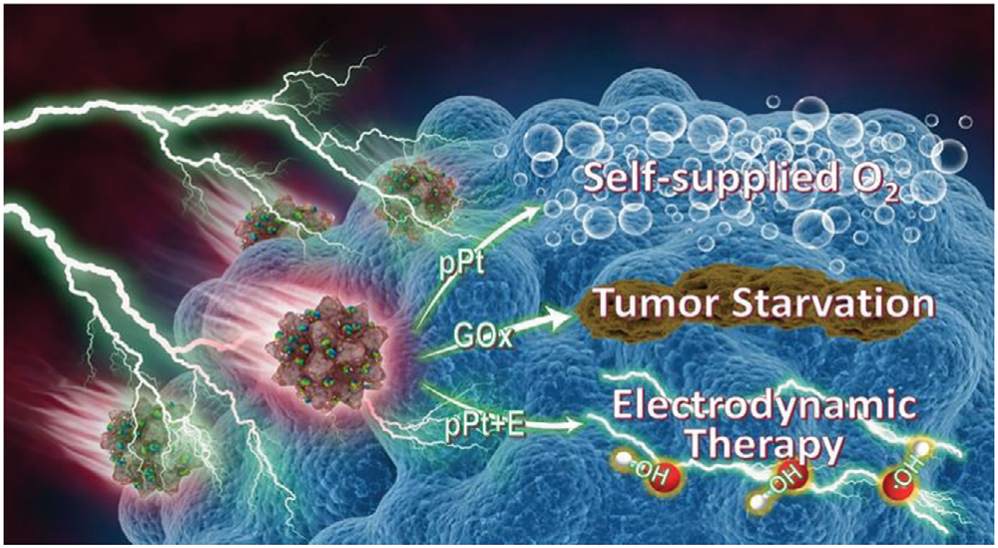
Illustration of functioning mechanism of pPt@GOx (PtG).

Here in this study, uniform platinum nanospheres with a porous microstructure (pPt) are synthesized and incorporated with GOx molecules (PtG) to enable synergistic EDT/starvation therapy with hypoxia modulation. As demonstrated in **Scheme** **1**, after being uptaken by tumor cells, GOx released from PtG catalyzes the oxidation of intracellular glucose to produce gluconic acid and H_2_O_2_, while pPt replenishes oxygen by triggering the decomposition of both endogenous and exogenous H_2_O_2_. The O_2_ supplied in turn facilitates the glucose consumption by GOx to promote tumor starvation. Meanwhile, pPts induce a considerable ROS production under an alternating electric field, leading to oxidative stress injury of cancer cells following an EDT mechanism. In consequence, significant in vitro and in vivo anticancer effect is achieved by PtG nanoparticles. This study, for the first time, offers a highly valuable therapeutic platform that may enable oxygen‐inductive starvation and EDT approaches in a synergistic manner for effective tumor ablation.

## 2 Results and Discussion

### 2.1 Synthesis and Characterization

**Figure 1:**
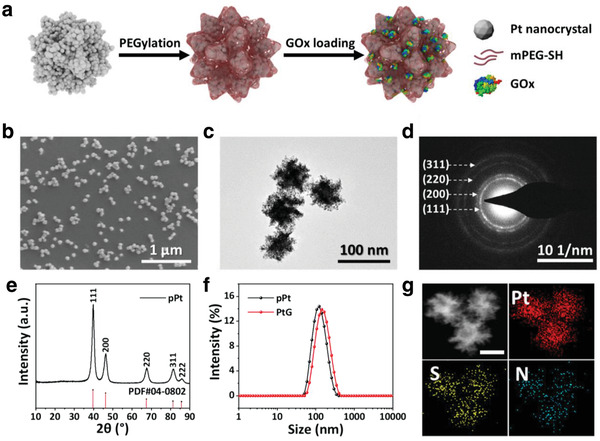
a) Illustration of the synthetic procedure of PtGs. b) Scanning electron microscope (SEM) image of pPts. c) Typical transmission electron microscope (TEM) image and d) corresponding SAED pattern of pPts. e) XRD patterns of pPts. f) DLS analysis of pPts and PtGs in PBS. g) Energy dispersive spectroscopy (EDS) element mapping of PtGs (scale bar: 50 nm).

The pPts were synthesized following a facile one‐step method by reducing H_2_PtCl_6_ with ascorbic acid in the presence of a Pluronic F127 surfactant and KBr.^[^ ^20^ ^]^ After surface functionalization using methoxy polyethylene glycol sulfhydryl (mPEG‐SH), GOx molecules were loaded on pPts owing to their porous microstructure, as illustrated in **Figure** **1**a. The as‐prepared pPts are of uniform spherical geometry with a dimension of ≈70 nm (Figure 1b). At a higher magnification, pPts present to be assembled by nanocrystals with a diameter of ≈5 nm (Figure 1c), favoring the formation of pores at the particle surface. The selected‐area electron diffraction (SAED) pattern is composed of concentric rings with bright spots, indicating the polycrystalline nature of pPts (Figure 1d). The obtained X‐ray diffraction (XRD) pattern exhibits a pure fcc platinum structure, which is consistent with the results of SAED pattern (Figure 1e). The concentration of pPt solutions was determined using inductively coupled plasma mass spectrometry (ICP‐MS) and UV–vis spectrometry (Figure S1, Supporting Information).

To improve the stability of pPts in aqueous solution, mPEG‐SH was used for surface modification by virtue of the affinity of platinum to thiol group. After surface modification, a narrow size distribution of pPts, with an average hydrodynamic diameter of 115 nm, presents according to the dynamic light scattering (DLS) analysis (Figure 1f). Subsequently, GOx was integrated on pPt, forming pPt@GOx nanoparticles (PtG). The concentration of GOx in PtG was examined by Bradford protein assay kit, indicating the GOx loading efficacy was ≈2.81%. The procedure of PEGylation and GOx loading did not induce clear variations in the microstructure of nanoparticles (Figure S2, Supporting Information). The zeta potential of PEGylated pPt and PtG are −16.1 and −18.1 mV, respectively (Figure S3, Supporting Information). The porous structure of pPt and PtG was verified by the N_2_ adsorption–desorption test (Figure S4, Supporting Information). It is clear that both Brunauer, Emmett and Teller (BET) surface area and Barrett, Joyner, and Halenda (BJH) pore volume decreased after the loading of GOx. The element mapping exhibits homogeneous distributions of Pt, S, and N in PtG, which indicates the successful grafting of mPEG‐SH and loading of GOx molecules (Figure 1g; Figure S5, Supporting Information). The loading of GOx leads to minor increase in the hydrodynamic diameter to ≈136 nm, without exerting any negative effect on the dispersivity of PtG particles. In addition, good stability and dispersivity of PtGs maintain in H_2_O, phosphate buffered saline (PBS), and RMPI‐1640 solutions, paving the way to the further assessments (Figure S6, Supporting Information).

### 2.2 The Electric‐Field‐Driven ROS Production by pPts

**Figure 2:**
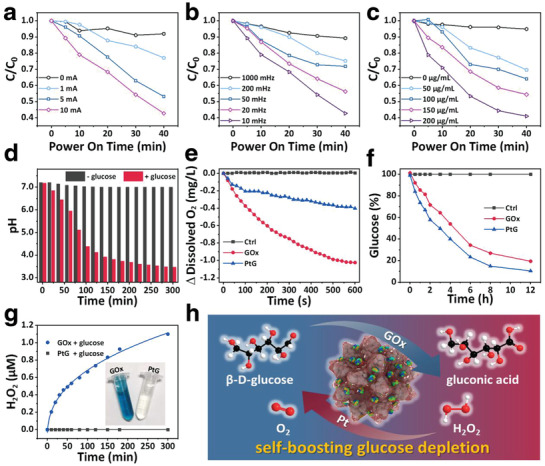
Degradation of MB in pPt solutions with varied a) current intensities, b) electric frequencies, and c) pPt concentrations. d) The variation of pH in PtG solutions with or without glucose. e) Oxygen and f) glucose consumption kinetics of GOx and PtGs solutions. g) H_2_O_2_ generation of PtGs and GOx in glucose solution (inset: photograph of solutions after chromogenic reaction). h) Schematic diagram of the self‐boosting glucose depletion of PtGs.

In order to investigate the electrocatalytic activity, methylene blue (MB) was used to evaluate the ROS production by pPts in the presence of a square‐wave electric field, following the procedure reported previously.^[^ ^18^ ^]^ As shown in **Figure** **2**a, the degradation of MB is high corresponding to the electric current applied to the reaction vessel (Figure S7, Supporting Information). At 0 mA electric current, a minor decrease in MB absorbance presents, which is attributed to the adsorption by the porous structure of pPts. When the current intensity is increased from 0 to 10 mA, the absorbance of MB decreases in a more rapid fashion, and the decline is positively correlated with the current intensity. Similarly, accelerated MB degradation occurs when the input frequency decreased from 1000 to 10 mHz (Figure 2b; Figure S8, Supporting Information) or increased pPts concentration, as expected (Figure 2c; Figure S9, Supporting Information). Overall, the ROS induction of pPts under an alternating electric field is highly dependent on its concentration, powering on time, current intensity, and frequency. The findings reflect that the porous microstructure of pPts prepared in this study does not induce any negative effect to the electrodynamic performance of Pt nanoparticles reported earlier.^[^ ^18^ ^]^ It is noteworthy that the loading of GOx molecules in PtG hardly induces clear adverse effects on its electrocatalytic activity (Figure S10, Supporting Information).

### 2.3 Self‐Boosting Glucose Depletion of PtG

The pH variation of PtG in PBS is examined in the presence and the absence of glucose, respectively. It is clear that, upon the addition of glucose, the pH of the PtG solution shows a significant decline (Figure 2d), implying that glucose is oxidized into gluconic acid by PtG. Meanwhile, the variations of glucose, oxygen, and hydrogen peroxide are investigated over time to compare the difference between PtG and free GOx (with identical GOx content). The findings reveal that PtG induces less oxygen consumption (Figure 2e) but more rapid glucose depletion over time comparing to that of GOx during the reaction (Figure 2f; Figure S11, Supporting Information). More interestingly, during the entire catalytic reaction, there is no clear sign for the H_2_O_2_ production for PtG, while considerable content of H_2_O_2_ is generated during the glucose consumption by free GOx (Figure 2g; Figure S12a, Supporting Information). This is attributed to the fact that pPt presents excellent catalytic ability to transform the H_2_O_2_ induced by GOx into O_2_ in a rapid fashion (Figure S12b, Supporting Information), and the O_2_ induced in turn facilitates the glucose consumption by GOx.

Overall, the mechanism of glucose depletion by PtG becomes clear. As demonstrated in Figure 2h, GOx molecules loaded on the nanoparticles catalyze the oxidation of glucose to generate gluconic acid and H_2_O_2_. The H_2_O_2_ produced is decomposed by pPt particles, inducing water and oxygen. It is worth noting that, in addition to the exogenous H_2_O_2_ induced during the reaction of glucose depletion, the endogenous H_2_O_2_, with an elevated magnitude in tumor tissue,^[^ ^21^ ^]^ can also be catalyzed by pPt to provide a certain content of oxygen. Owing to the crucial role of oxygen played in the GOx catalytic reaction, the oxygen compensation ability of pPt can considerably promote the glucose consumption by GOx under a hypoxia condition. The reaction loop, enabled by PtG, implements the self‐boosting glucose depletion process, paving the way to its unique performance in inducing tumor starvation.

### 2.4 In Vitro Study

**Figure 3:**
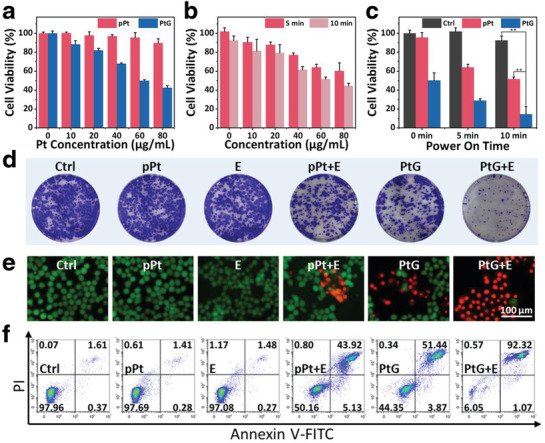
In vitro cytotoxicity assay. a) 4T1 cells viabilities treated with varied concentrations of pPts and PtGs for 24 h. b) Relative cell viabilities of 4T1 cells treated with pPts after agitated by a square‐wave current (5 mA, 10 mHz) for 5 or 10 min. c) Synergistic cell‐killing effect of PtGs with an electric field. *p*‐values: **p < 0.01. d–f) Corresponding colony formation, Live&Dead staining images, and quantitative flow cytometry analysis of cell apoptosis/necrosis of 4T1 cells treated with all seven groups.

First, the cytotoxicity of pPt and PtG was examined and compared via a standard cell counting kit‐8 (CCK‐8) assay. As shown in **Figure** **3**a, no clear toxicity to 4T1 cells was induced by pPt up to 80 µg mL^−1^, while the PtG group exhibited a remarkable inhibition effect to cellular viability. With the increased glucose concentration in the culture medium, the inhibition effect of PtG was enhanced, reflecting that its killing effect is mainly attributed to the cellular starvation induced by GOx (Figure S13, Supporting Information). Subsequently, to examine the EDT effect of pPts, 4T1 cells were exposed to a square‐wave electric field, set at 5 mA and 10 mHz for 5 min, after being incubated with pPt for 4 h. The cell viabilities declined continuously with increasing concentration of pPt. In addition, when the treatment duration was extended to 10 min, the decrease in cell viabilities accelerated accordingly, as expected (Figure 3b). It was clear that 4T1 cells treated with PtG under the agitation of an electric field (PtG+E) presented the most significant enhancement in cellular inhibition, comparing to the group of pPt plus electric field (pPt+E) and PtG alone (Figure 3c). The colony formation assay exhibited similar results that the PtG+E group enabled the strongest suppression to cell survival and proliferation comparing to other groups (Figure 3d). The most striking red fluorescence appeared in the PtG+E group according to the Live&Dead cell staining assay, verifying the findings above (Figure 3e; Figure S14, Supporting Information). To examine the cellular apoptosis in a more quantitative manner, the cells were stained with annexin V‐FITC and propidium iodide (PI) to examine apoptotic and necrotic cells (Figure 3f). Approximately 93% of cells were apoptosis in the PtG+E group, while PtG and pPt+E groups showed ≈55% and ≈49% cellular apoptosis, respectively. The results are in good agreement with the cell viability assay.

**Figure 4:**
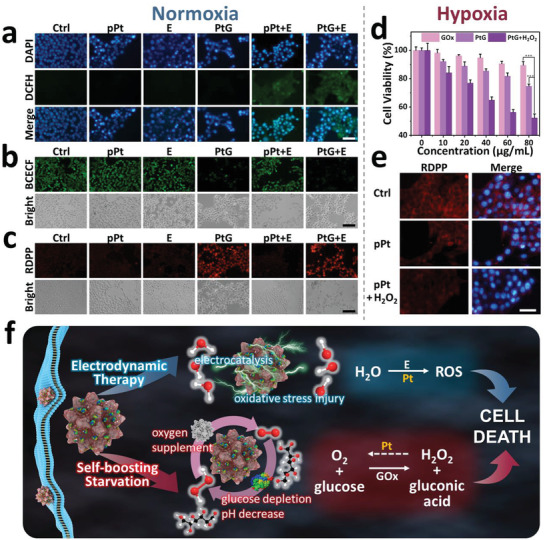
Cellular abnormalities induced by electric field and starvation. Fluorescence images of a) intracellular ROS (scale bar: 100 µm), b) pH (scale bar: 200 µm), and c) oxygen (scale bar: 200 µm) in 4T1 cells cultured with different sample groups under a normoxia condition. d) Relative cell viabilities of 4T1 cells incubated with free GOx, PtGs, and PtGs+H_2_O_2_ under a hypoxic condition for 24 h. *p*‐values: ***p < 0.001. e) RDPP fluorescence in 4T1 cells treated with pPts in the absence and presence of H_2_O_2_ under a hypoxic condition (scale bar: 100 µm). f) Mechanism illustration of inhibition effect induced by PtGs.

To uncover the in vitro antitumor mechanism of PtG under the square‐wave electric field, 2,7‐dichlorofluorescein diacetate (DCFH‐DA) probe was used to detect the presence of intracellular ROS (**Figure** **4**a). Cells treated with pPt+E and PtG+E showed an elevated level of ROS generation, as expected, while no clear fluorescence was presented in the PtG group. This indicates that pPts induce significant content of intracellular ROS under the square‐wave electric field. Meanwhile, the intracellular pH and oxygen were assessed to examine the catalytic reaction triggered by GOx loaded on PtG within cells. As detected using 2′,7′‐bis‐(2‐carboxyethyl)‐5‐(and‐6)‐carboxyfluorescein, acetoxymethyl ester (BCECF AM), only PtG‐ and PtG+E‐treated cells presented remarkably weakened green fluorescence, indicating that the pH decline was induced by GOx (Figure 4b). Subsequently, when the hypoxia indicator, [Ru(dpp)_3_]Cl_2_ (RDPP), was used for intracellular oxygen examination, bright red fluorescence was observed in PtG and PtG+E groups comparing to other sample groups, owing to the considerable oxygen depletion occurred (Figure 4c). It is worth noting that EDT treatment presents a negligible effect on intracellular pH and oxygen level. In addition, GOx was labeled with fluorescein isothiocyanate (FITC) to prepare fluorescent nanocomposites PtG_FITC_. and the strong green fluorescence in the cells treated with PtG_FITC_ indicates the presence of GOx released intracellularly (Figures S15 and S16, Supporting Information).

To illustrate the favoring role of pPt played in the GOx‐mediated starvation, the viability of cells cultured with PtG was assessed with comparison of free GOx under a hypoxia condition. Meanwhile, H_2_O_2_ (100 × 10^−6^ m) was added during cell culture to simulate the tumorous microenvironment (Figure 4d; Figure S17, Supporting Information). Without sufficient oxygen supply, free GOx showed limited cytotoxicity, but PtG presented clear cellular inhibition effect. When H_2_O_2_ was induced, this inhibition by PtG was remarkably promoted. In addition, the intracellular oxygen was examined using RDPP after cell culture with pPts (without loading GOx) the hypoxia atmosphere. Strong fluorescence was observed in the control group, whereas only a faint glow was observed when cells were incubated with pPt and H_2_O_2_ (Figure 4e). The findings indicate that the oxygen replenishment is effectively induced intracellularly by pPts in the presence of H_2_O_2_.

Overall, the full picture of phenomena, induced by PtG after cellular uptake, is now visible. As demonstrated in Figure 4f, the unique catalytic reaction loop triggered by PtG occurs within cancer cells, and significant glucose consumption is induced to enable the considerable cellular starvation and inhibition. In parallel, pPt, under the alternating electric field, triggers the decomposition of water molecules to generate ROS, following the mechanism reported previously.^[^ ^18^ ^]^ The excessive content of intracellular ROS induces cellular oxidative stress, and further promotes apoptosis of cancer cells. pPt, here in the synergistic approach, serves not only as an EDT agent, but also an amplifier in promoting GOx‐mediated starvation via oxygen induction.

### 2.5 In Vivo Antitumor Efficacy

**Figure 5:**
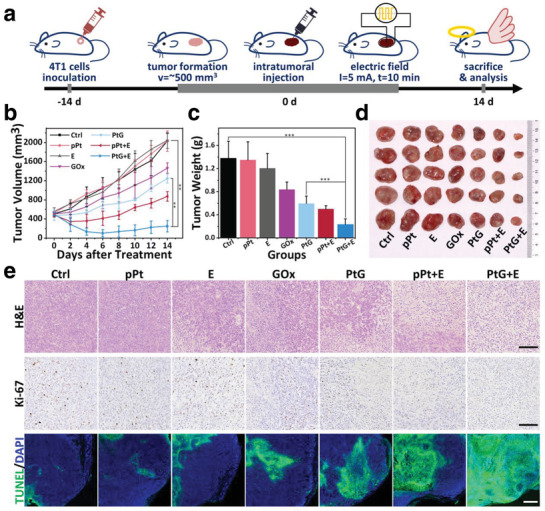
In vivo antitumor efficacy. a) Demonstration of the treatment process. b) Variation of tumor volume after different treatments by all sample groups (*n* = 5). c) Average tumor weight on day 14 after various treatments. d) Photographs of tumors harvested from different treatment groups. e) H&E staining (scale bar: 100 µm), Ki‐67 staining (scale bar: 100 µm), and TUNEL staining (scale bar: 500 µm) images of tumor tissue collected from different mice groups 24 h after treatments. *p*‐values: ***p < 0.001, **p < 0.01.

The in vivo antitumor activity of PtGs under square‐wave electrical stimulation was evaluated in a mouse model, following the treatment process demonstrated in **Figure** **5**a. After 2 weeks since subcutaneous inoculation of 4T1 cancer cells, the tumor volume reached ≈500 mm^3^ in average. The mice were then randomly divided into seven groups (*n* = 5): 1) untreated, 2) pPt, 3) only square‐wave electric field (E), 4) free GOx, 5) PtG, 6) pPt plus square‐wave electric field (pPt+E), and 7) PtG plus square‐wave electric field (PtG+E). After intratumoral injection, the square‐wave electric stimulation was applied at 5 mA for 10 min (Figure S18, Supporting Information), and the variation of tumors in mice was examined in the following 14 days. In addition, the in vivo biodistribution was evaluated at 24 h postinjection (Figure S19, Supporting Information). The distributed amount of Pt within tumor reached ≈40.9 µg g^−1^, while negligible presence of Pt was observed in other major organs.

While no clear changes were observed in body weight for all the seven groups (Figure S20, Supporting Information), the tumor growth differed significantly in all seven mice groups (Figure 5b; Figure S21, Supporting Information). The volume of tumors grew continuously in Groups 1–3, whereas a moderate growth inhibition was observed among the tumors treated with free GOx (Group 4), PtG (Group 5), and pPt+E (Group 6). More specifically, compared with PtG, the inhibition efficacy of free GOx was slightly weaker. It is attributed to the fact that free GOx is more likely to be deactivated and metabolized, while pPt can provide certain protection for GOx from enzymatic degradation in the complex physiological microenvironment. Meanwhile, pPt can enhance the starving effect induced by GOx via the modulation of hypoxia. An apparent ablation was observed when tumors were treated with pPt+E, which is ascribed to the ROS generation following an EDT approach. Overall, the most significant inhibition of tumor growth is presented in Group 7, owing to the synergistic effects from EDT and starvation, as expected. On day 14, the tumors were excised, photographed, and weighed (Figure 5c,d). The results validated that the treatment of PtG+E enabled the most potent tumor inhibition.

Additionally, hematoxylin and eosin (H&E) staining, Ki‐67 staining, and immunofluorescence terminal deoxynucleotidyl transferase‐mediated dUTP‐biotin nick end labeling (TUNEL) were conducted for histological analysis after mice scarification (Figure 5e). In the H&E analysis, no clear abnormal area was observed in Groups 1 and 2. Comparing to the minor damage caused in the tumor tissue by Groups 3–6, the treatment of PtG+E induced the most severe destruction, manifesting as the ubiquitous nuclear pyknosis, karyolysis, vacuolation, and membrane rupture. Moreover, the most pervasive green fluorescence in TUNEL assay and the lowest percentage of Ki‐67‐positive cells also corroborated that PtG+E treatment led to the most significant apoptosis and highest inhibition level of cell proliferation (Figure S22, Supporting Information). The findings in histological examinations confirm the antitumor effectiveness induced by PtG under an alternating electric field, as expected.

Overall, it is worth noting that, compared to the current electrochemical therapy (EChT) which has been used clinically, the electrode configuration has been significantly simplified in EDT due to its distinctive functioning mechanisms. However, the operation procedure of EDT may still remain inconvenient in the actual clinical practice. To develop EDT‐based therapeutic protocol with less invasive electrodes following its fundamental principle is therefore anticipated to tackle this challenge in the follow‐on investigations.

## 3 Conclusions

Here for the first time, this study proposed a feasible therapeutic platform enabling synergistic multimodal approach, electrodynamic therapy combined with self‐boosting tumor starvation. For this purpose, pPts with fine microstructure were synthesized and incorporated with GOx molecules (PtG). GOx can effectively consume glucose via catalytic oxidation and generate H_2_O_2_. Apart from serving as a delivery cargo, pPt catalyzes both endogenous and exogenous H_2_O_2_ to produce O_2_, which in turn magnifies the GOx‐mediated starvation. Meanwhile, under an alternating electric field pPt can also induce a considerable content of intracellular ROS via electrodynamic catalytic reaction to promote the cellular apoptosis. In consequence, the PtG nanocomposite exhibits remarkable synergistic curative effect toward malignant solid tumor both in vitro and in vivo. This study has therefore demonstrated a highly potential therapeutic strategy and its feasible platform for effective tumor treatment.

## Conflict of Interest

The authors declare no conflict of interest.

## Supporting information

Supporting Information
