# Supplementary material for: Porous Pt Nanospheres Incorporated with GOx to Enable Synergistic Oxygen‐Inductive Starvation/Electrodynamic Tumor Therapy
Source: Adv Sci (Weinh). 2020 Jul 6;7(17):2001223. doi: 10.1002/advs.202001223 (PMC7507307; doi:10.1002/advs.202001223)
Supplement: Supplementary file 1 — Supporting Information [file ADVS-7-2001223-s001.pdf]

Copyright WILEY-VCH Verlag GmbH & Co. KGaA, 69469 Weinheim, Germany, 2020.

Supporting Information

Porous Pt nanospheres incorporated with GOx to enable synergistic oxygen-inductive starvation/electrodynamic tumor therapy

Zijie Lu, JiaYu Gao, Chao Fang, Yi Zhou, Xiang Li, and Gaorong Han**

Z.J. Lu, C. Fang, Dr. X. Li, Prof. G.R. Han
State Key Laboratory of Silicon Materials
School of Materials Science and Engineering
Zhejiang University
Hangzhou, Zhejiang 310027, P. R. China
E-mail: xiang.li@zju.edu.cn; hgr@zju.edu.cn

J.Y. Gao, Dr. Y. Zhou
The Affiliated Stomatology Hospital, Zhejiang University School of Medicine
Key Laboratory of Oral Biomedical Research of Zhejiang Province
Hangzhou, Zhejiang 310027, P. R. China

Z.L. and J.G. contributed equally to this work.

Experimental section

Materials

Chloroplatinic acid hexahydrate ($H_2PtCl_6·6H_2O$), Methylene blue (MB) were purchased from Aladdin. Ascorbic acid (AA, 99%), Potassium bromide (KBr, 99%), methoxy polyethylene glycol sulfhydryl (mPEG-SH, Mw=5000) were obtained from Macklin. Pluronic F-127, tris(2,2'-bipyridyl)dichlororuthenium(II) hexahydrate ($Ru(dpp)_3Cl_2·6H_2O$, 99.95%), 2',7'-dichlorofluorescein diacetate (DCFH-DA, 97%), fluorescein isothiocyanate (FITC, 95%) were acquired from Sigma-Aldrich. Glucose oxidase was purchased from Sangon Biotech. Molecular probes, 4',6-Diamidino-2-phenylindole (DAPI), calcein acetoxymethyl ester (calcein-AM), propidium iodide (PI), 2',7'-bis-(2-carboxyethyl)-5-(and-6)-carboxyfluorescein,acetoxymethyl ester (BCECF AM), Bradford protein assay kit, cell

counting kit-8 (CCK-8), annexin V-FITC and propidium iodide apoptosis detection kit, crystal violet staining solution were obtained from Beyotime Biotechnology.

Characterization

The sample morphology was examined using a field-emission scanning electron microscopy (FESEM, Hitachi SU-70). Transmission electron microscope (TEM) imaging and elemental mapping were acquired using FEI Tecnai F20 TEM. The crystalline phase identification was determined by an X-ray diffraction instrument (RIGAKU D/MAX 2550/PC). The zeta potential and dynamic light scattering (DLS) size distribution of samples were measured by a Malvern zetasizer (Nano-ZS). The nitrogen adsorption-desorption isotherms were measured by an automatic surface area and porosity analyzer (Tristar 3020, Micromeritics). Inductively coupled plasma mass spectrometry (ICP-MS, PerkinElmer NexION300X) was adopted for quantifying the Pt concentration. The absorbance spectra were measured by a spectrophotometer (Shimadzu, UV-2700). The fluorescent images were observed under a fluorescence inversion microscope (Nexcope, NIB900). The cell apoptosis and necrosis was determined by a flow cytometry (Beckman Coulter, CytoFLEX LX). The images of H&E and Ki-67 staining was obtained with a digital slide scanner (Hamamatsu, NanoZoomer 2.0-RS), and the TUNEL images were acquired by a digital slide scanner (Olympus, VS120).

Synthesis of porous Pt nanosphere incorporated with GOx (PtG)

Porous platinum nanospheres (pPts) were synthesized following a one-pot method. Typically, 25 mg Pluronic F-127 was added to 2.5 mL aqueous solution of 0.84 mM KBr. 1 mL 40 mM H_2PtCl_6 aqueous solution and 2.5 mL 0.14 M ascorbic acid solution were added into the mixture dropwise. The solution was stirred in a 70℃ oil bath for 12 h. Subsequently, the mixture was cooled down to ambient temperature and centrifuged at 12000 rpm for 10 min. In order to remove the residual Pluronic F-127, the collected sample was washed repeatedly with ethanol and water. Then, methoxy PEG sulfhydryl (mPEG-SH, Mw = 5000)

was used surface modification of pPts. Briefly, 1 mg pPts and 5 mg mPEG-SH were mixed in 5 mL ultra-pure water under ultrasonication for five minutes. After 48 h PEGylation process, the PEGylated pPts were collected by centrifugation and washed with water for three times. Finally, to prepare the GOx functionalized nanoplatform, GOx and the sample were dispersed in water and stirred overnight. Finally, the final sample was obtained by centrifugation.

Evaluation of electrodynamic activity

The experimental instruments were arranged following the previous literature. A function signal generator was employed as the power source to provide electric output. The electro-catalytic activity was assessed by measuring the degradation of methylene blue. Briefly, different amounts of pPts were added to 2 mL PBS containing 15 μM MB. The function signal generator was connected, and a square-wave electrical signal with certain voltage and frequency was applied. The current was monitored and adjusted with the aid of a multimeter. After powering on for different period (0, 5, 10, 20, 30 and 40 min), 50μL of the mixture was collected and diluted with PBS to 200 μL, and the UV-vis absorbance spectra of MB were collected.

Catalytic abilities of PtGs

To evaluate the catalytic ability of PtG, the pH changes of PtG in PBS were initially examined, both in the presence or the absence of glucose. Glucose (1 mg/mL) was added to PBS solution containing PtG under stirring. The pH value was monitored by a pH meter.

In order to explore the role of pPt played in the enzymatic activity of GOx, PBS solutions containing 1 mg/mL glucose were treated with PtG or free GOx. At different time intervals, the solutions were collected to measure the glucose and H_2O_2 concentration. The dissolved oxygen content was monitored by an oxygen meter.

The glucose-consuming ability of PtG was evaluated through DNS method. Briefly, glucose (1 mg/mL) was dissolved into 10 mL phosphate-buffered saline, and then PtGs and GOx (1 μg/mL GOx) were added respectively. At particular time points, 0.5 mL solution was

extracted and mixed with 1.5 mL DNS solution. The mixture was heated in boiling water for 5 min, followed by quenching in room temperature water for another 20 min. The UV-vis absorbance spectra was detected and the absorbance of each sample at 600 nm was measured to quantify the concentration of glucose.

The H_2O_2 concentration was determined through a colorimetric method with horseradish peroxidase (HRP) and TMB. Briefly, glucose was added into PBS solution with PtG or free GOx, respectively. At predetermined time points, 200 μL of the solution was collected and mixed with 300 μL HRP (1 U/mL), 1.5 mL PBS (pH = 5.8) and 300 μL TMB (2 mM) ethanol solution. The peak UV-vis absorbance of ox-TMB was recorded to calculate the variation of H_2O_2 concentration.

The dissolved oxygen content was measured by a dissolved oxygen meter. The procedure was similar to that of the glucose consumption to compare PtG and free GOx. In addition, to verify the oxygen compensation ability of pPts in presence of GOx and glucose, the dissolved oxygen content was also measured after adding H_2O_2 to the pPt aqueous solution.

Cell culture

4T1 cancer cells were cultured in RPMI-1640 medium with 10% fetal bovine serum and 1% penicillin/streptomycin at 37°C in a humidified 5% CO_2 atmosphere. To create the hypoxic and normoxic culture condition, the oxygen content of incubator was set as 2% and 20%, respectively.

***In vitro* cytotoxicity assay**

For cytotoxicity assay, 4T1 cells were seeded in 96-well plate with a density of 10^4 cells per well for 24 h. Subsequently, the medium was replaced with fresh RMPI-1640, followed by addition of pPt, PtG and free GOx with vaired concentrations. After 24 h incubation, the supernatant was removed, and fresh medium containing 10% CCK-8 was added to each well. After further incubation for 1h, the absorbance of each well was measured on a microplate reader at 450 nm.

To evaluate the starving therapeutic effect of PtG, the cells were seeded, and the culture medium was replaced with fresh RPMI-1640 medium of different glucose content. Subsequently, cells were co-cultured with gradient concentrations of PtG. After 24 hours, the relative cell viabilities were measured *via* standard CCK-8 assay.

For EDT treatment, 4T1 cells were seeded in 24-well plates for 24 h. Then, the same concentration of pPt and PtG (60 μg/mL) were added to the media and cultured for 4h. A function signal generator was connected with the cells in series. Using a multimeter, the electric current was set at 5 mA for 10 min. After 24 h, a standard CCK-8 assay was carried out to evaluate the relative cell viabilities.

To further investigate the self-boosting effect induced by pPt for GOx-mediated starvation, the cell viability test was assessed both in normoxic (20% O_2) and hypoxic (2% O_2) conditions. First, the 4T1 cells were seeded in a 96-well plate and incubated in a normoxic environment overnight. Afterwards, PtG and free GOx were added to the cells. In addition, to simulate the microenvironment, additional PtG with 100 μM H_2O_2 was added and culture for further 24 h. The cell viability was assessed by CCK-8 assay.

Colony formation assay

4T1 cells were seeded at ~2000 cells per well of a 6-well plate. After incubated for 24 h, the culture medium was replaced by RPMI-1640 medium containing pPt and PtG for 4 h, followed by EDT treatment. The untreated cells were set as the control. Then, the culture medium was removed and replaced with fresh RPMI-1640 medium. After 10~14 days, cells were washed with PBS twice, immobilized with 4% methanol for 10 min, and stained with crystal violet for 15 min at room temperature. Following rinsing with ultra-pure water, each well with formed colonies was photographed.

Live & Dead cell staining assay

4T1 cells were seeded in 6-well plates and cultured overnight, followed by treated with PBS, pPt and PtG, respectively. After 4h, the cells received EDT treatment and incubated

overnight. The untreated cells were set as the control. Then, all cells were stained by Calcein-AM and PI solutions and incubated at 37℃ for 30 min. The dye solutions were removed, and each well was washed with PBS for three times. Finally, the images were observed under fluorescence inversion microscope.

Cell apoptosis analysis

The cell apoptosis was assessed by flow cytometry. The cells were seeded in 24-well plates and incubated for 24 h. Then, cells were treated with PBS, pPt and PtG for 4 h, followed by EDT treatment. The untreated cells were set as the control. After 24 h, the cells were digested with trypsin and collected by centrifugation. After repeatedly washing, the cells were co-stained with Annexin V-FITC and PI for 20 min, followed by examination using a flow cytometry to determine the ratio of apoptotic and necrotic cells.

Synthesis of fluorescein isothiocyanate (FITC) labeled GOx (GOx-FITC)

The GOx-FITC was prepared following the known approach. Typically, 25 μL of FITC in DMSO solution (5 mg/mL) was mixed with 1 mL sodium carbonate-sodium bicarbonate buffer solution (pH 9.4) containing 10 mg/mL GOx, and stirred at 4 °C in the dark for 12h. Subsequently, the solution was dialyzed in deionized water overnight in dark, followed by lyophilization.

Cellular uptake

To investigate the cellular internalization of the nanocomposites, 4T1 cells were seeded in 6-well plates and cultured for 24h, the medium was replaced with fresh medium containing pPt or PtG_{FITC}. After four hours, the culture medium was removed, and the cells were washed with PBS before examination using fluorescence inversion microscope.

GOx release profile analysis

The GOx concentration in PtG and the release profile were determined with Bradford assay kit. Typically, 20 μL GOx containing supernatant was collected and mixed with 200 μL

Bradford solution. The absorbance of the solution was measured on a microplate reader at 560 nm.

Intracellular ROS detection

4T1 cells were seeded in 6-well plates and incubated for 24h, followed by the addition of pPt and PtG. After 4h, cells were stained with 20 μM DCFH-DA, and co-cultured for 30 min. The cells were then treated with electric field at 5 mA for 10 min and incubated at 37℃ for 1h. Cells incubated with PBS but without electric field were used as the control. After washing with PBS, the cells were fixed by 4% methanol for 10 min. The cell nuclei were stained with DAPI for 10 min at room temperature, followed by three-time washing with PBS. Finally, DCFH fluorescence was observed with fluorescence inversion microscope.

Intracellular pH and oxygen evaluation

4T1 cells were seeded in 6-well plates and incubated overnight. For intracellular pH detection, the cells were treated with PBS, pPt and PtG, followed by EDT treatment. The cells treated without samples and electric field were set as control. After 6~10 hours, the cells were stained with 5 μM BCECF-AM for 1 h, followed by observation using fluorescence inversion microscope.

For intracellular oxygen detection, the cells were seeded in 6-well plates. After incubation overnight, the culture medium was replaced by fresh RPMI−1640 medium containing 5 μM $[Ru(dpp)_3]Cl_2$. After 4h, the medium was removed, and the cells were rinsed with PBS for three times. Then, PBS, pPt and PtG were added and co-incubated under a normoxic condition for 4 h, followed by EDT treatment. After 6~10 hours, the cells were washed with PBS before fluorescence imaging. In addition, to verify the intracellular oxygen generation ability of pPt, the cells were incubated with pPt under hypoxic condition, while leaving the rest of the procedures remained unchanged.

***In vivo* antitumor efficacy**

All animal procedures were performed in accordance with the Guidelines for Care and Use of Laboratory Animals of Zhejiang University and approved by the Animal Ethics Committee of Zhejiang University. To obtain the tumor-bearing mice, four-weeks-old female BALB/c mice were subcutaneously inoculated with 50 μL PBS suspension of 4T1 cells. When the tumor reached ~500 mm^3 in average size (14 days after inoculation), the mice were randomly divided into seven groups and treated respectively with: (1) PBS, (2) pPt, (3) PBS plus electric field, (4) free GOx, (5) PtG, (6) pPt plus electric field, (7) PtG plus electric field. The aqueous solutions (50 μL) of pPt, GOx and PtG were injected intratumorally, and the corresponding Pt concentration is 2 mg/mL. After 10 min, the tumor sites (Group 3, 6, 7) were exposed to 5 mA square wave electric field for 10 min. Since the day that the mice received treatment, their body weight and tumor volume were recorded every two days, up to 14 days. The diameter of tumors were measured with a digital caliper in two dimensions. The volume was calculated according to the following equation: tumor volume V (mm^3) = $(W^2 \times L)/2$, where width W is the shorter dimension of tumor and length L is the longer dimension perpendicular to W. On day 14, all the mice were sacrificed, and tumors were excised, photographed and weighed. And the contents of Pt element in the main organs and tumor were examined by inductively coupled plasma-mass spectrometry (ICP-MS).

For histology analysis, tumors from each group were harvested 24 hours after the treatment. Then, the tumor tissues were sliced and fixed in formalin and sectioned. Tissue slices were stained with H&E, Ki-67, and TUNEL before the examination using fluorescence microscopy.

Supporting Figure List

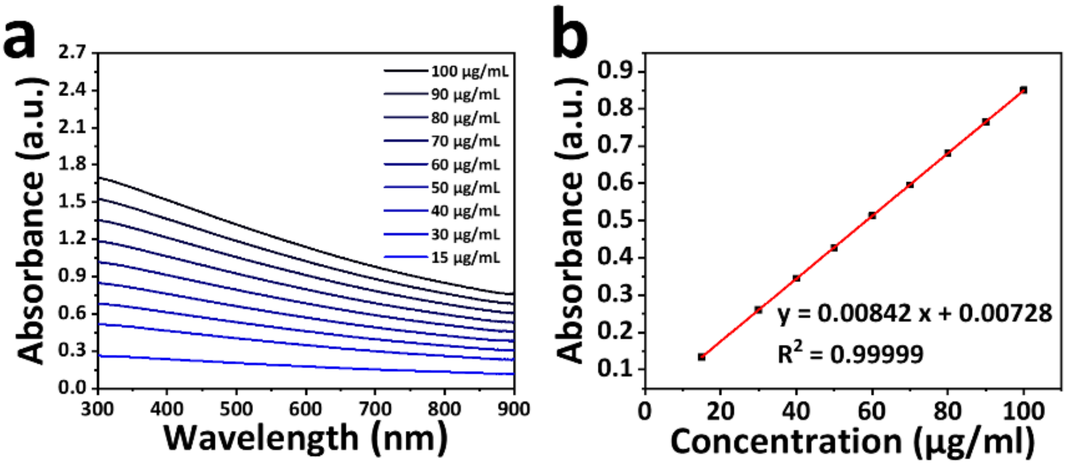

Figure S1. a) UV-vis absorption spectra of pPts with different concentrations measured by ICP-MS, and b) the accordingly calculated standard curve of absorbance at 800 nm.

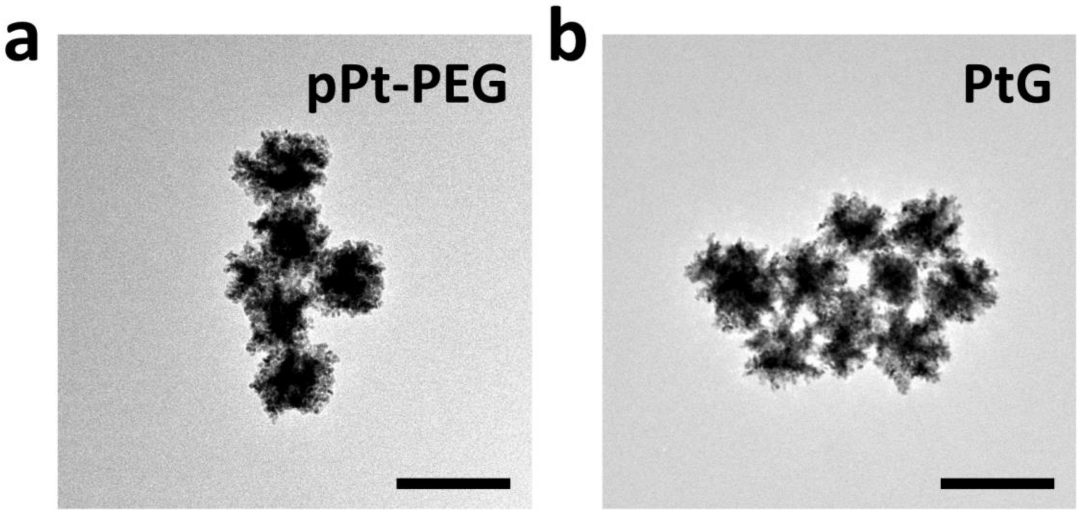

Figure S2. TEM images of a) PEGylated pPts and b) PtGs (scale bar: 100 nm).

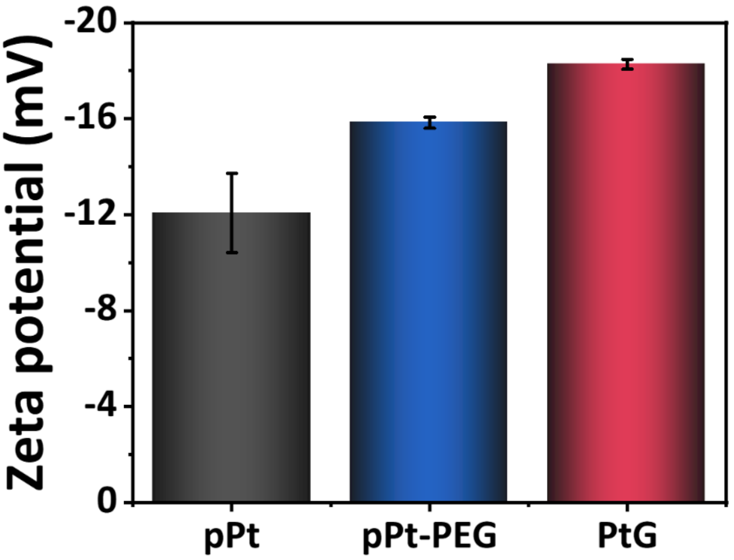

Figure S3. Zeta potential of pPt, PEGylated pPt and PtG.

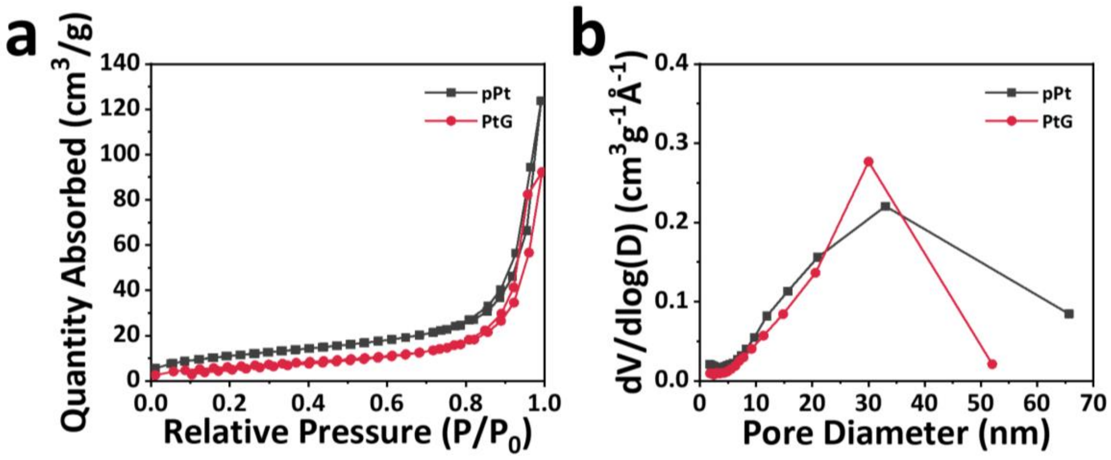

c

	BET surface area (m^2/g)	BJH pore volume (cm^3/g)
pPt	~40.3	~0.191
PtG	~23.5	~0.143

Figure S4. a) Nitrogen adsorption-desorption isotherms and b) the pore size distribution of PEGylated pPts and PtGs; c) Summary of Brunauer-Emmett-Teller (BET) surface area and the Barrett-Joyner-Halenda (BJH) pore volume of PEGylated pPt and PtG.

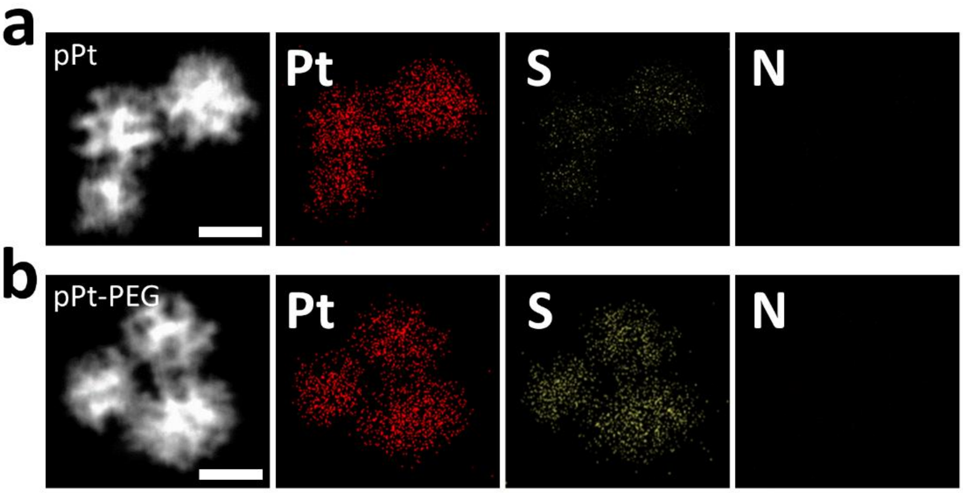

Figure S5. EDS element mapping of a) pPts and b) PEGylated pPts (scale bar: 50 nm).

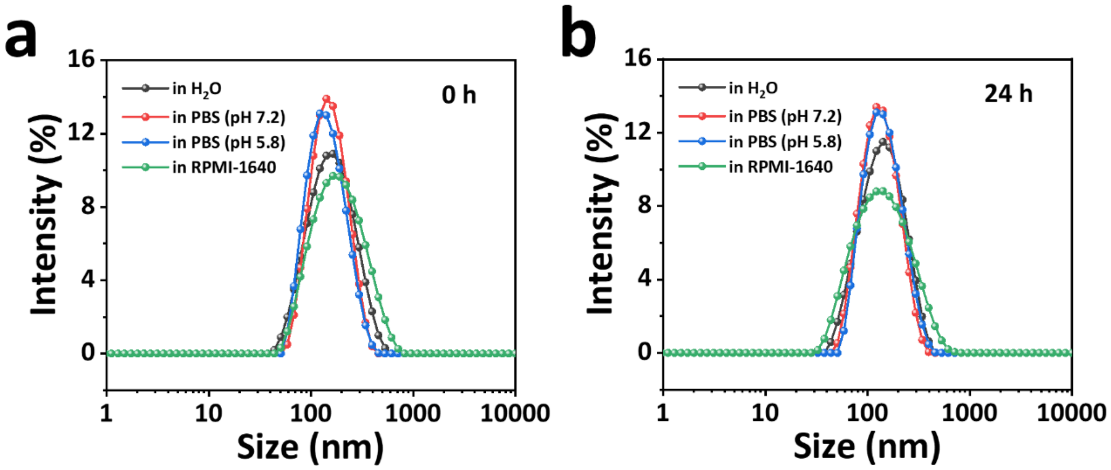

Figure S6. The DLS analysis of PtGs in H_2O, PBS (pH 7.2 and 5.8), and RMPI-1640 at 0h and 24h.

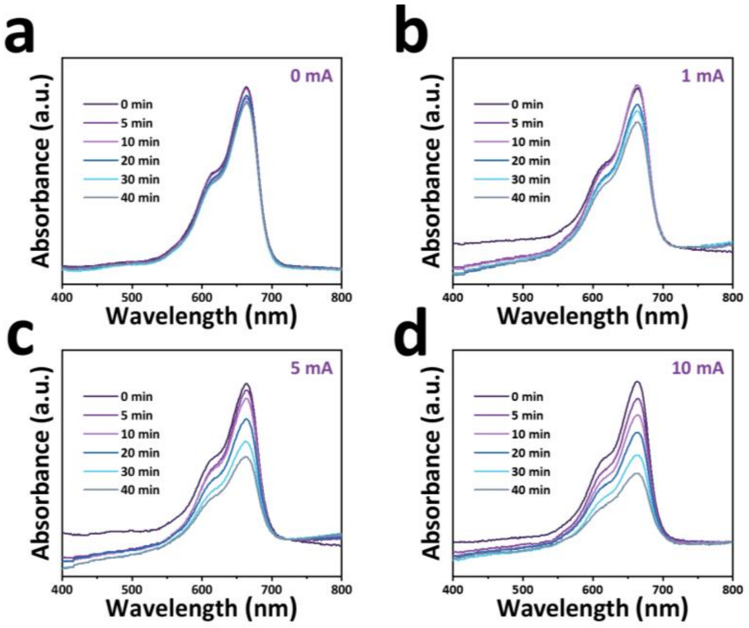

Figure S7. UV-vis spectra of MB solutions containing 200 μg/mL pPt under a square-wave electric field (10 mHz) with various current intensities for different time of period.

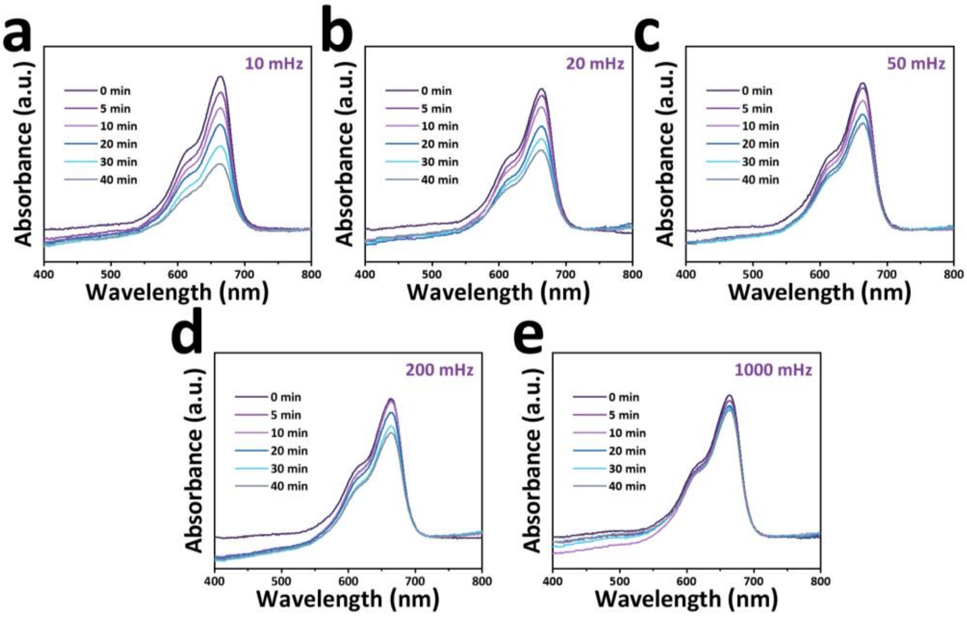

Figure S8. UV-vis spectra of MB solutions containing 200 μg/mL pPt under a square-wave electric field (5 mA) with various input frequencies for different time of period.

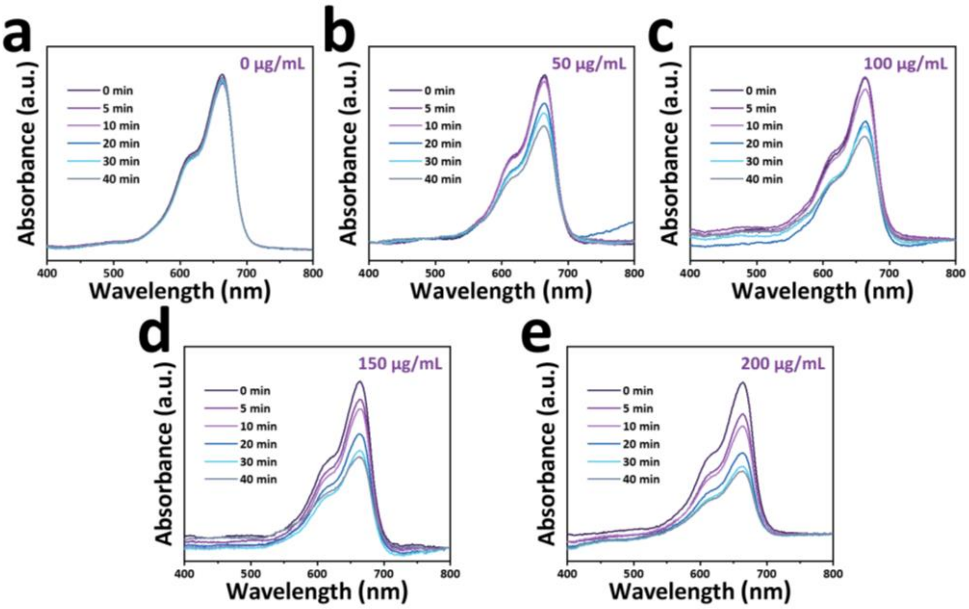

Figure S9. UV-vis spectra of MB solutions containing pPt with different concentrations under a square-wave electric field (5 mA, 10 mHz).

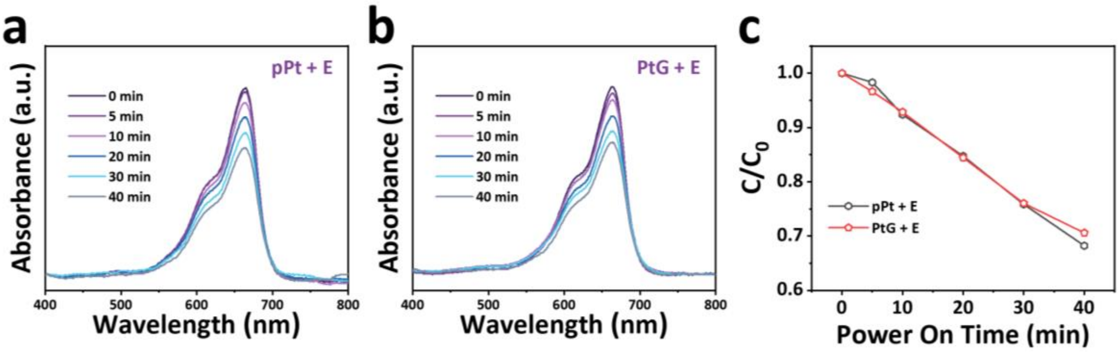

Figure S10. Degradation of MB induced by a) pPt and b) PtG with identical Pt content (100 µg/mL), and c) the quantitative comparison of the eletro-catalytic activity of two samples.

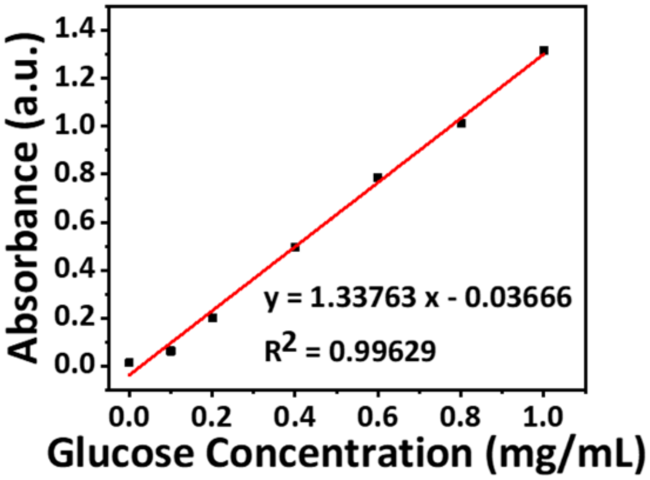

Figure S11. The standard curve of the UV-vis absorbance at 600 nm of DNS solution with different glucose concentrations.

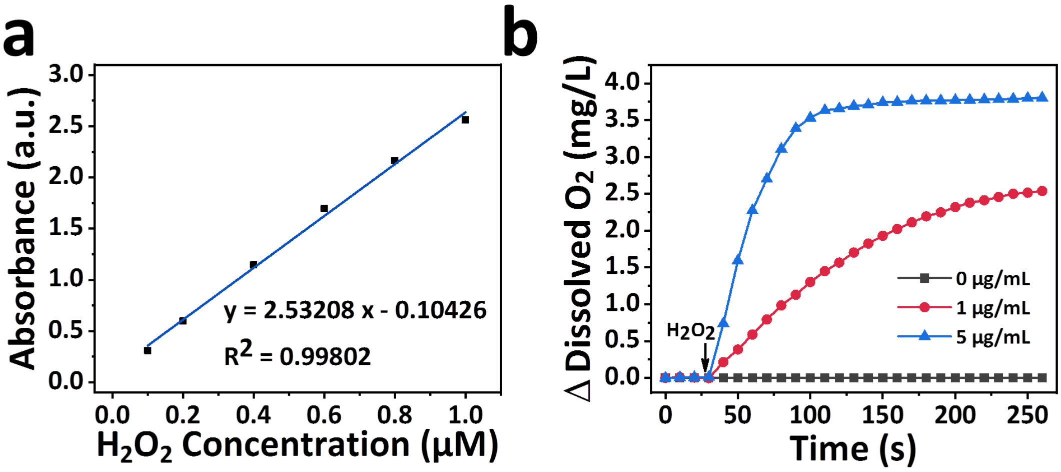

Figure S12. a) The standard curve of varied concentration of H_2O_2 oxidized TMB at 652 nm in the presence of horseradish peroxidase. b) Oxygen-generation ability of different concentrations of pPts with addition of H_2O_2.

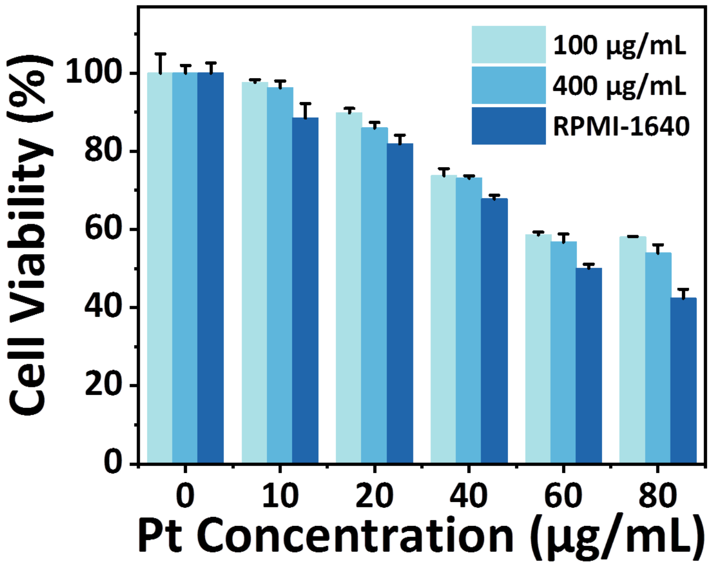

Figure S13. Relative viabilities of 4T1 cells incubated with varied concentrations of PtG in culture medium of different glucose content.

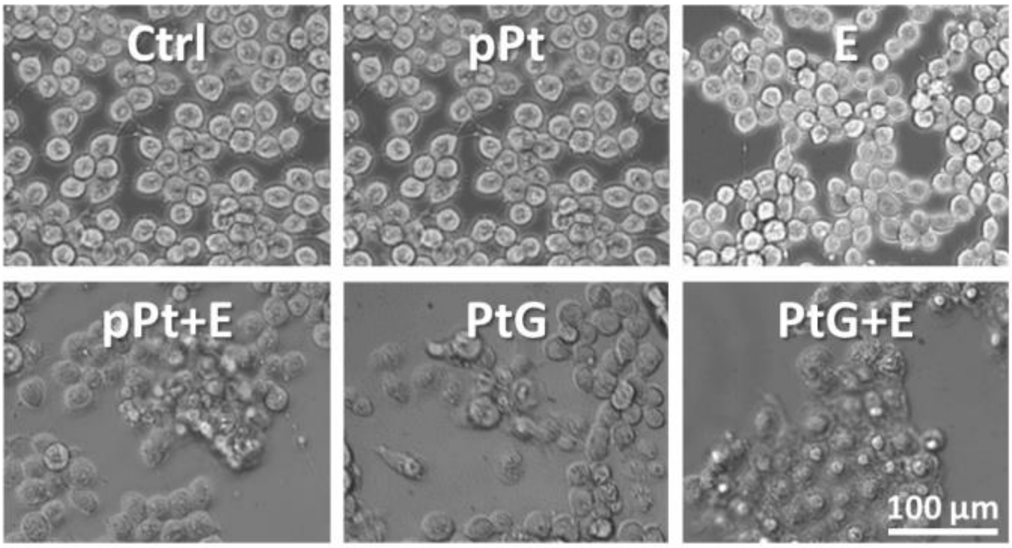

Figure S14. The corresponding bright field images of Live&Dead stained 4T1 cells from different groups.

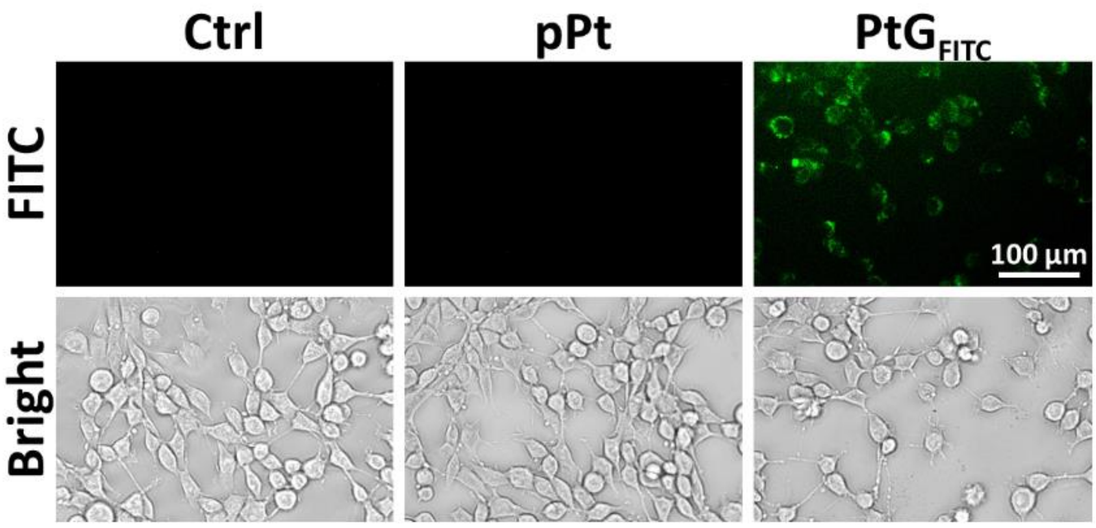

Figure S15. The fluorescence and bright field images of 4T1 cells after being co-cultured with PBS, pPt and PtG_{FITC} for 4 h.

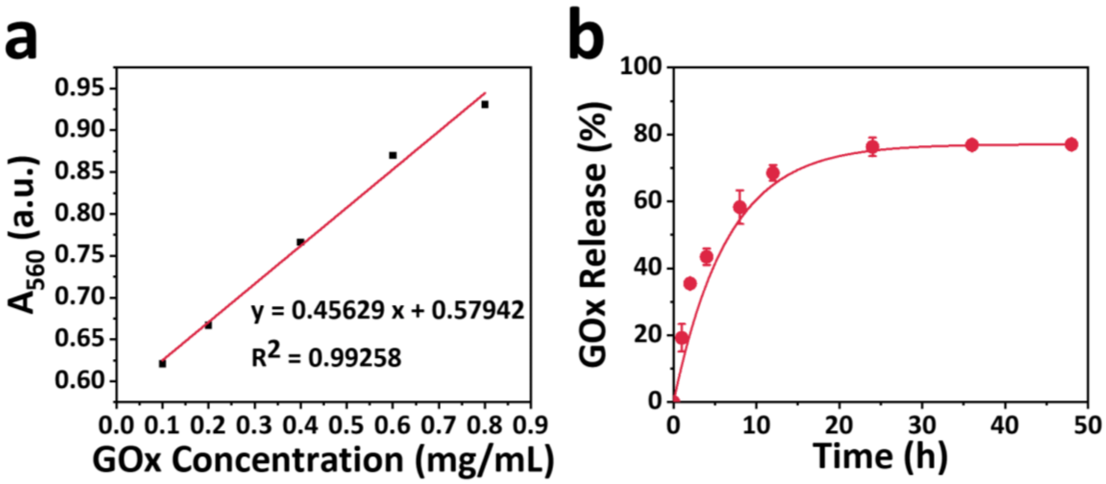

Figure S16. a) The concentration-dependent standard curve of GOx by Bradford assay. b) The release profile of GOx from PtG determined by Bradford assay.

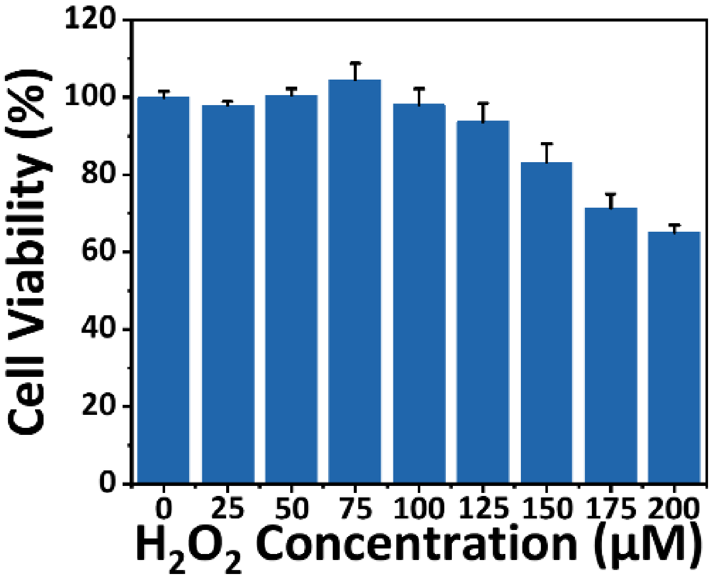

Figure S17. Relative cell viabilities of 4T1 cells incubated with H_2O_2 solutions with varied concentrations.

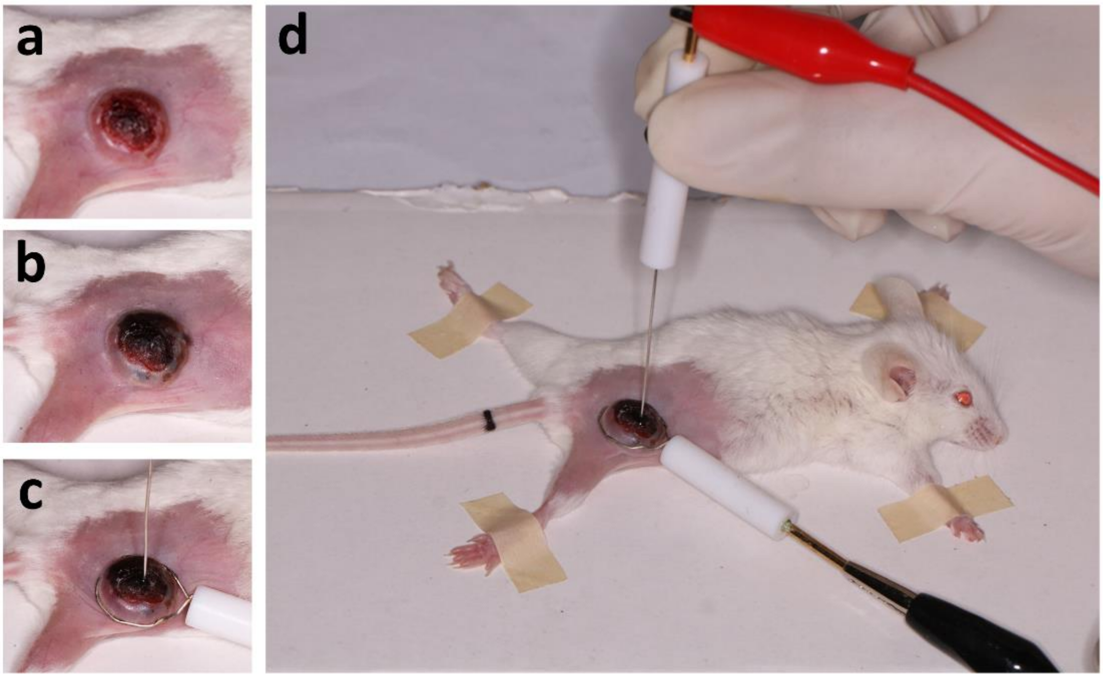

Figure S18. Photographs for operational procedure of EDT.

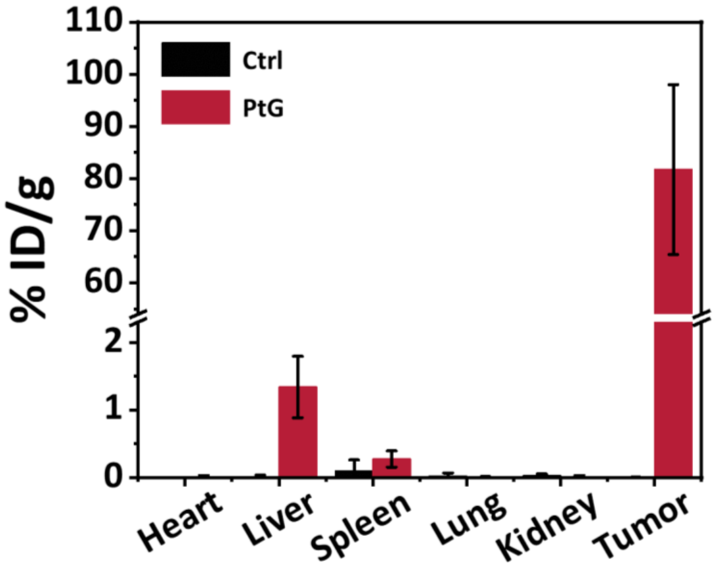

Figure S19. The biodistribution of PtG (% injected dose (ID) of Pt per gram of tissues) in main organs and tumor at 24h post-injection.

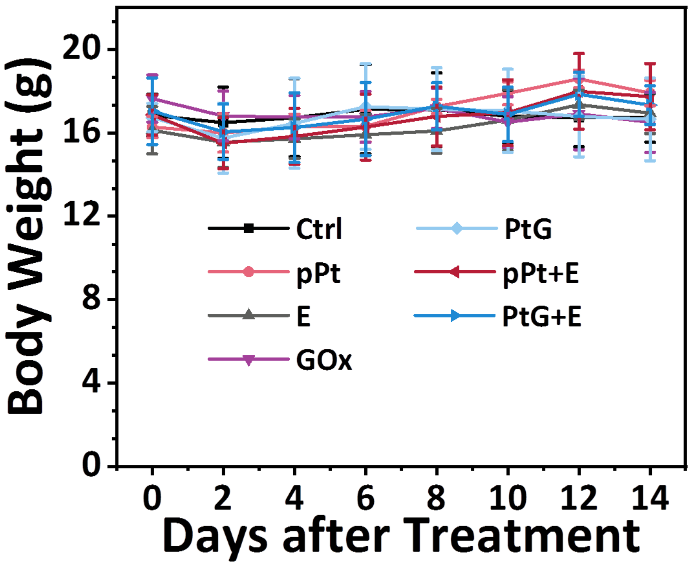

Figure S20. Body weight of mice in 14 days after the indicated treatments

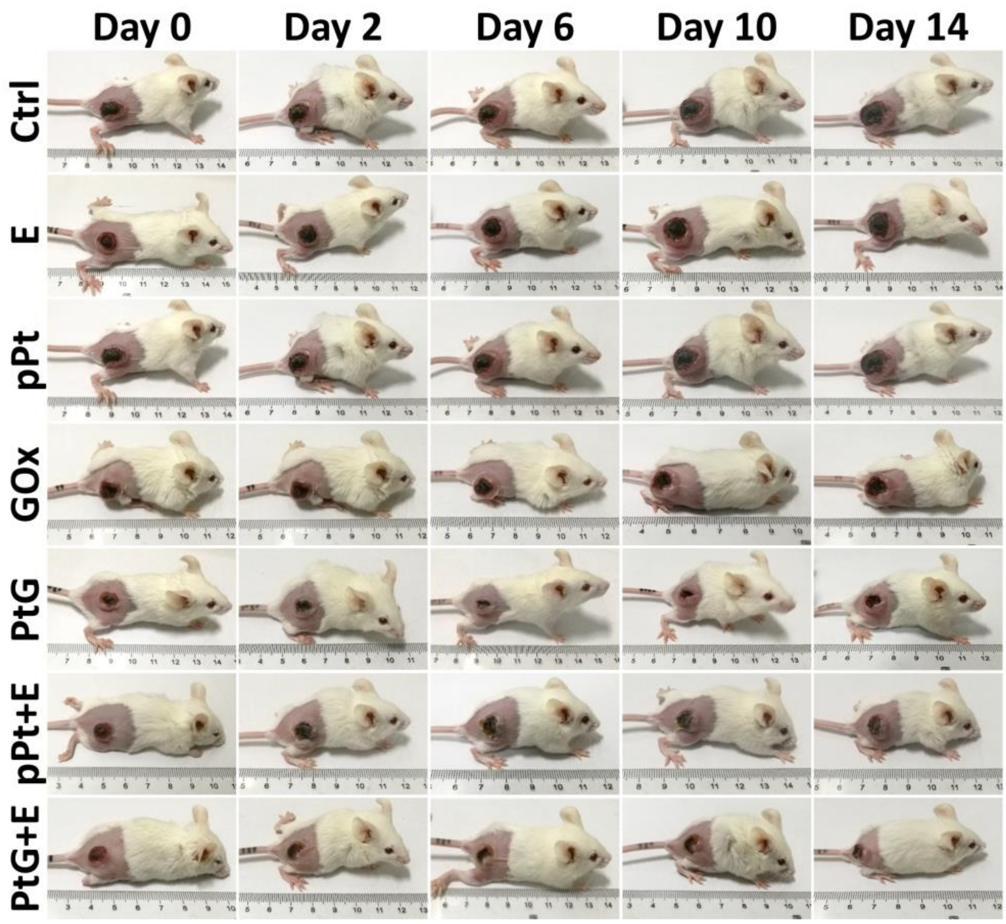

Figure S21. Photographs of tumor-bearing mice after different treatments.

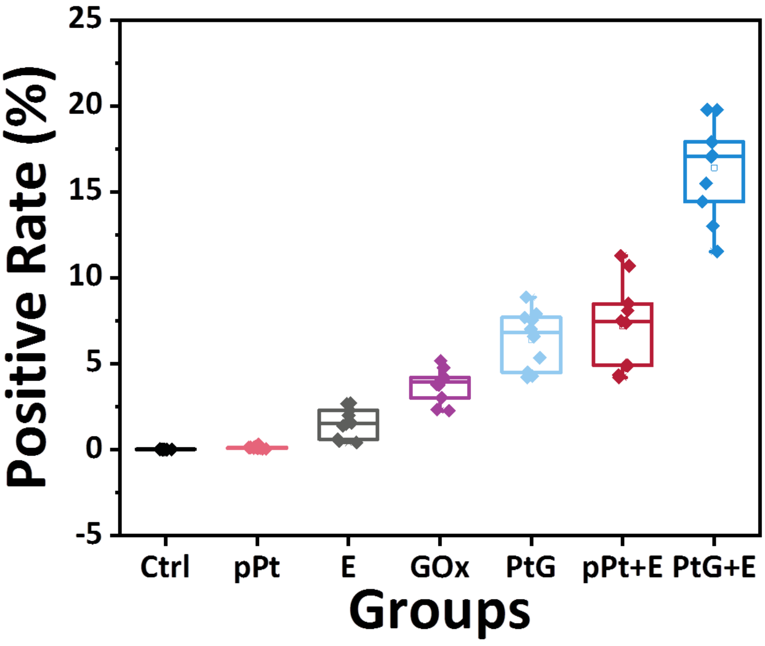

Figure S22. Statistical analysis of TUNEL staining images
